# Comparison of the efficacy of rivaroxaban and dabigatran etexilate in preventing venous thrombosis after arthroplasty: A protocol of randomized controlled trial

**DOI:** 10.1097/MD.0000000000023814

**Published:** 2021-01-08

**Authors:** Runze Song, Ruisheng Qu, Yu Wang, Dong Zhou, Anping Zhang

**Affiliations:** Department of Vascular Surgery, Lanzhou University Second Hospital, Lanzhou 730030, Gansu Province, China.

**Keywords:** dabigatran etexilate, joint replacement, randomized controlled trial, rivaroxaban, venous thromboembolism

## Abstract

**Background:**

New oral anticoagulants (NOAC) are gradually accepted by clinical practice for its convenient route of administration and stable effect. Both rivaroxaban and dabigatran etexilate have been used in the prevention and treatment of venous embolism after arthroplasty, but there is a lack of direct comparison between the 2 effects. Therefore, the purpose of this randomized controlled trial was to evaluate the efficacy and safety of 2 new oral anticoagulants, rivaroxaban, and dabigatran etexilate, in the prevention of venous thromboembolism after joint replacement.

**Methods:**

This is a prospective randomized controlled trial to study the efficacy and safety of rivaroxaban and dabigatran etexilate in the prevention of venous thromboembolism after joint replacement, and is approved by the clinical research ethics of our hospital. Patients were randomly divided into 1 of 2 treatment regimens:

(A) rivaroxaban oral group and
(B) dabigatran etexilate oral group.

Patients, doctors, nurses, and data collection assistants were blinded to group allocation. The indicators of observation include:

(1) validity indicators: asymptomatic deep venous thrombosis (DVT), symptomatic DVT, symptomatic pulmonary embolism (PE) incidence, all-cause mortality;
(2) safety indicators: incidence of major bleeding and clinically related non-major bleeding events and other adverse events.

Data were analyzed using the statistical software package SPSS version 25.0 (Chicago, IL).

**Discussion:**

This study will evaluate the efficacy and safety of rivaroxaban and dabigatran etexilate in preventing venous thrombosis after joint replacement. The results of this experiment will provide clinical basis for the use of rivaroxaban or dabigatran etexilate to prevent venous thrombosis after joint replacement.

**Ethics and dissemination:**

Private information from individuals will not be published. This systematic review also does not involve endangering participant rights. Ethical approval was not required. The results may be published in a peer-reviewed journal or disseminated at relevant conferences.

**OSF Registration number:**

DOI 10.17605/OSF.IO/QVDCW.

## 1 Introduction

Venous thromboembolism (VTE), one of the most common complications after joint surgery, which includes deep venous thrombosis (DVT) and pulmonary embolism (PE), is a complication that orthopedic surgeons must focus on its prevention and pay attention after surgery.^[1]^ The study showed that patients who did not receive medication had a 40% to 60% incidence of deep venous thrombosis and a 1% to 2% risk of pulmonary embolism.^[2]^ For the prevention of VTE, in addition to the commonly used physical prevention methods (such as the lower limb power pump to enhance muscle contraction), oral antithrombotic, and diffusion drugs are also one of the basic prevention methods, and have been recommended by domestic and foreign clinical practice guidelines.^[3,4]^

At present, the common anticoagulants in drug prevention are vitamin K antagonists (VKA), unfractionated heparin (UFH), low molecular weight heparin (LMWH), new oral anticoagulants (NOAC), etc.^[5]^ The enoxaparin, which represent the LMWH, is a classic drug to prevent VTE after major orthopedic surgery, but it is inconvenient for patients because of the daily subcutaneous injection.^[6]^ Therefore, in recent years, the new oral anticoagulant has attracted the attention of clinicians because of its high specificity and delicious clothing. NOAC include Xa factor inhibitors (e.g., rivaroxaban) and direct thrombin inhibitors (e.g., dabigatran etexilate), which have more advantages in their delivery routes, for anticoagulation will not be affected by food intake, and have little effect on interactions with other drugs, so there is no need for regular monitoring during medication. It is stable, and gradually accepted clinically.^[7,8]^

At present, rivaroxaban and dabigatran etexilate have been widely used in the prevention and treatment of venous embolism after joint replacement, and their clinical effects have been confirmed. However, few studies have directly compared the efficacy and safety of the 2 in the prevention and treatment of venous embolism after arthroplasty. Therefore, the purpose of this randomized controlled trial is to evaluate the efficacy and safety of 2 new oral anticoagulants, rivaroxaban, and dabigatran etexilate in the prevention of venous thromboembolism after joint replacement.

## 2 Materials and methods

### 2.1 Protocol register

**Figure 1:**
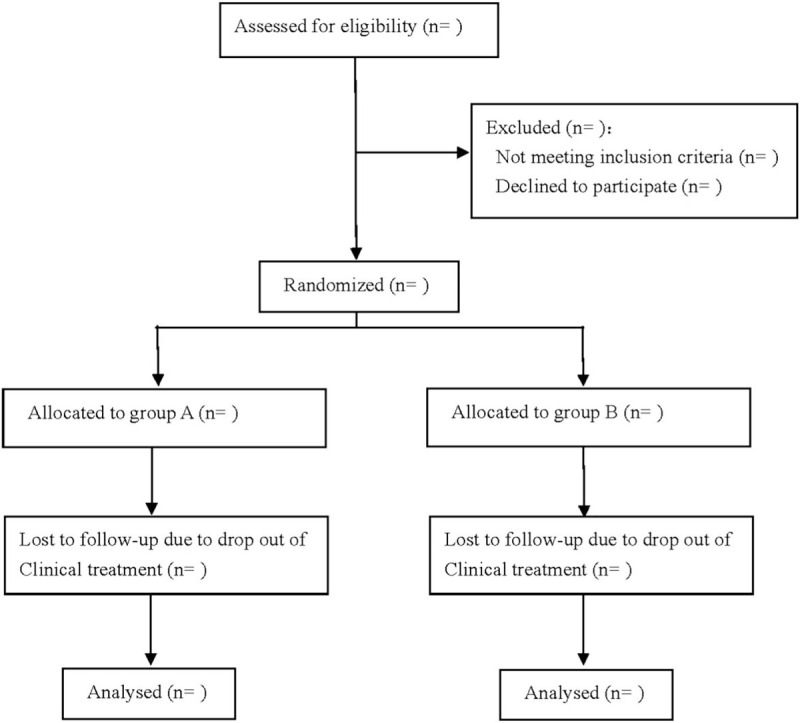
Flow diagram.

This is a prospective randomized controlled trial to investigate the efficacy and safety of rivaroxaban and dabigatran in the prevention of venous thrombosis after joint replacement. We followed the Consolidated Standards of Reporting Trials (CONSORT) guidelines for reporting randomized trials and provided a CONSORT flow diagram (Fig. 1) and the Standard Protocol Items: Recommendations for Interventional Trials (SPIRIT) 2013 statement.

### 2.2 Ethics and registration

This research program is in accordance with the Helsinki Declaration and approved by the Clinical Research Ethics Committee of our hospital. This protocol has been registered in open Science Framework (OSF) (Registration number: DOI 10.17605/OSF.IO/QVDCW). All patients need to sign a written informed consent before they are randomly assigned to continue the trial.

### 2.3 Sample size

The aim of this prospective study was to explore a comparison of the efficacy and safety of rivaroxaban and dabigatran etexilate in preventing venous thrombosis after joint replacement. The exploratory of this study does not necessarily require formal sample size calculations. So, based on past clinical experience and previous studies,^[9]^ the sample size of 600 participants (300 in each group) was sufficient to achieve the actual purpose of the trial.

### 2.4 Patients

Inclusion criteria: meet the criteria for elective joint replacement surgery (including shoulder, hip, knee, including trauma, or arthritis); no signs of embolism in venous color Doppler ultrasonography and pulmonary CT examination; all patients are the first time to underwent joint replacement.

Exclusion criteria:

(1) existence of severe coagulation dysfunction;
(2) existence of cardiovascular and cerebrovascular diseases;
(3) allergic to the drugs used in this experiment;
(4) existence of severe immune dysfunction;
(5) tumor or bone tuberculosis;
(6) amalgamation of other systemic major diseases;
(7) existence of other new oral anticoagulants contraindication;
(8) unable to understand the research protocol after explanation or unwilling to participate.

### 2.5 Study design

Eligible participants were randomly assigned to the rivaroxaban oral group or dabigatran etexilate oral group in a 1:1 ratio using a central network-based random tool. The random sequences are generated by SAS 9.3 software (SAS Institute, Cary, NC) by independent statisticians who do not participate in trial implementation or statistical analysis. Randomization was performed without any stratification. The clinical research coordinator entered participant information on the tablet and was given a random number. The research assistant obtained the allocation of participants from the computer. Throughout the study, the research assistant was responsible for screening, recruiting participants, and assigning random numbers to participants who had been included. Results evaluator was responsible for the evaluation of the scale. The grouping of personnel is unknown to all clinician, researchers, research assistants, participants, intervention supervisors, and statisticians who conduct statistical analysis. The drugs were distributed by unwitting nurses to patients with corresponding numbers according to the drug number.

### 2.6 Interventions

Before operation, the patients were examined by Doppler ultrasound to determine whether there was venous embolism, and if necessary, additional pulmonary CT was taken to rule out pulmonary embolism. The rivaroxaban group began to take 10 mg, once a day, 6 to 8 hours after operation, while the dabigatran etexilate group began to take 1 to 4 hours after operation, which was halved at the first time, and recovered to 220 mg once a day the next day. Both course of treatments lasted for 2 weeks.

Ultrasonic Doppler examination of limbs was performed by ultrasound doctors at 1 or 2 weeks after operation. If the formation of DVT is suspected, ultrasonic Doppler examination should be performed immediately; if pulmonary embolism is suspected, pulmonary CT or angiography should be performed in addition to ultrasonic Doppler examination, and the results should be recorded. The patients were followed up for 30 to 35 days after the last administration. Hematological examination was performed before operation, on the same day after operation and before discharge, and abnormal laboratory indexes were monitored. Hematological and imaging tests could also be performed during follow-up if necessary.

### 2.7 Outcome measures

(1) Validity index: The primary outcome is a combination of total VTE (venography or symptomatic DVT and/or PE) and all-cause mortality during treatment, which is defined as the time from the date of the first study to 3 days after the last administration, and the secondary outcome is a combination of major VTE (venography or symptomatic proximal DVT and/or PE) and VTE-related mortality during treatment.
(2) Security index: The main safety outcome is the incidence of massive hemorrhage from the first medication in the study to 2 days after the last use (continued treatment). Massive hemorrhage is defined as clinically fatal bleeding that occurs in critical organs (such as retroperitoneal, intracranial, intraocular, or intraspinal), requiring surgery, outside the surgical site, and associated with it. The secondary safety outcome was the incidence of clinically related non-major bleeding events, defined as polyphyly hemorrhage, accidental hematoma (>25 cm^2^), excessive wound hematoma, epistaxis (>5 min), gingival bleeding (>5 min), gross hematuria, rectal bleeding, cough or hematemesis, vaginal bleeding, semen bleeding, traumatic intra-articular bleeding, or surgical site bleeding, etc. Secondary safety outcomes also include the incidence of adverse reactions, such as abnormal laboratory indicators (with special emphasis on liver and kidney function indicators), allergic reactions, and other adverse events.

### 2.8 Data collection and management

The whole process of data collection and recording will be carried out by 1 or 2 assistants. Personal information about potential participants and registered participants will be collected, shared, and stored in a separate storeroom to protect pre-, during, and post-trial confidentiality. The access to the database will be restricted to the researchers in this study team.

### 2.9 Statistical analysis

Data were analyzed using the statistical software package SPSS version 25.0 (Chicago, IL). Continuous variables were described as the mean ± standard deviation, and differences between groups were analyzed using a series of one-way analysis of variance (ANOVA) with Bonferroni's post-hoc test, while differences between groups over time were analyzed using multi-way ANOVA with Bonferroni's post-hoc test. Categorical variables were described as the number (%), and were analyzed by Fisher's exact test. A *P* value of <.05 was considered statistically significant.

## 3 Discussion

Although current preventive measures can play a good role in treatment, there is still a high incidence of VTE after hip replacement and knee replacement.^[10]^ Low molecular weight heparin is mostly used in the prevention of venous thrombosis, but daily subcutaneous injection brings inconvenience to patients. Therefore, in recent years, new anticoagulants have attracted much attention of clinicians because of their high specificity and oral characteristics.

National Institute for Health and Care Excellence (NICE) guidelines recommend rivaroxaban should start preventing at 6 to 12 hours postoperatively, total hip arthroplasty (THA) should prevent for 28 to 35 days, total knee arthroplasty (TKA) should prevent for 10 to 14 days.^[11]^ American College of Chest Physicians (ACCP) recommends rivaroxaban prophylaxis at 6 to 8 hours postoperatively in 2012, and the prophylaxis time for hip or knee arthroplasty should be no less than 10 to 14 days, which can be extended to 35 days.^[5]^ Rivaroxaban is a highly selective, oral direct factor Xa inhibitor. Inhibitor Xa can inhibit the endogenous and exogenous pathways of blood clotting waterfall, thus reduce the production of thrombin and inhibit thrombosis.^[12]^ Dabigatran etexilate belongs to β-alanine thrombin inhibitor, which is a new and synthetic direct thrombin inhibitor and the prodrug of dabigatran.^[13]^ The basic principle is that direct thrombin inhibitors bind to the activation sites of thrombin to inhibit thrombin and inhibit the conversion of fibrinogen to fibrin, as well as activating factor 5 (FVa), activating factor 8 (FVIIIa), activating factor 9 (FIXa), activating factor 13 (FXIIIa), and platelet kinase activating receptor, which then blocks the final steps of the clotting cascade mesh and thrombosis.^[14]^ In 2013, the European Medicines Agency have approved the phase III clinical trial of dabigatran etexilate for the prevention of venous thrombosis after hip or knee arthroplasty.

At present, as a new type of oral anticoagulant, rivaroxaban and dabigatran etexilate have been used in the prevention and treatment of postoperative venous thrombosis. Many countries have carried out randomized controlled trials compared with conventional anticoagulants injected with low molecular weight heparin,^[15–17]^ but there is no comparative study on the efficacy and safety of the 2. The results of this study will suggest clinical evidence comparing taking rivaroxaban with taking dabigatran etexilate orally by providing data about the changes in various measurements from the rigorously conducted. This study will provide a basis for clinicians to choose acupoint injection or sacral canal injection to treat LDH.

This study also has the following limitations: because this is a single-center randomized controlled study, there may be regionalization in the included population, and there may be a certain bias in the results; factors such as the age and cause of disease of the patients included in the study may affect the results to a certain extent.

## Author contributions

**Data curation:** Runze Song and Yu Wang.

**Funding acquisition:** Anping Zhang.

**Investigation:** Ruisheng Qu and Dong Zhou.

**Resources:** Ruisheng Qu and Dong Zhou.

**Software:** Ruisheng Qu and Dong Zhou.

**Supervision:** Anping Zhang.

**Writing – original draft:** Runze Song and Yu Wang.

**Writing – review & editing:** Anping Zhang.
